# The Relationship between Body Fat Percentage and Some Anthropometric and Physical Fitness Characteristics in Pre- and Peripubertal Boys

**DOI:** 10.3390/ijerph16071170

**Published:** 2019-04-01

**Authors:** Márta Szmodis, Iván Szmodis, Anna Farkas, Zsófia Mészáros, János Mészáros, Han C.G. Kemper

**Affiliations:** 1 Department of Health Sciences and Sport Medicine, University of Physical Education, Budapest 1123, Hungary; szmodis.ivan@gmail.com (I.S.); farkas@tf.hu (A.F.); dr.meszaros.zsofia@gmail.com (Z.M.); meszaros.zsofia@tf.hu (J.M.); 2 Amsterdam Public Health Research Institute, Amsterdam UMC, 1081 Amsterdam. The Netherlands; hancgkemper@upcmail.nl

**Keywords:** fat content, physique, physical fitness tests, 9–13-year-old boys, Hungary

## Abstract

The main aim of this study was to compare anthropometric and physical fitness indicators of boys of the same chronical age but with different fat percentages. Subjects were Hungarian boys aged 9–13 years (*N* = 6919). Anthropometry was measured according the guidelines of the International Biological Program. Relative body fat was estimated by Drinkwater–Ross’s method (1980); Conrad’s growth type of physique was also estimated (1963). Physical fitness was tested with 30 m dash (s), standing long jump (cm), fistball throw (m), and 1200 m run (s). Subjects of each cohort were grouped into seven subgroups with fat percentage ranges of 4%. Differences between subgroups were tested by one-way ANOVA. In the case of a significant *F*-test, Tukey’s post-hoc tests were used. The level of effective random error was set at 5% in all significance tests (*p* < 0.05). Except for the three groups with low fat percentages, values of body weight, stature, body mass index, and plastic and metric indexes were significantly higher; results of 30 m, 1200 m running, and standing long jump were worse in all groups with higher fat percentages. An interesting finding of the current study is that body fat percentage also influenced the physical fitness of non-overweight and obese children as well when using merely the 4% ranges in grouping by fatness. The lower the fat the better the physical fitness was in this sample of pre- and peripubertal boys.

## 1. Introduction

It is well known that, despite regional interventions, the incidence and prevalence of overweight and obesity among children is increasing in modern societies, including Hungary [1,2,3,4,5,6,7]. Obesity in childhood predicts obesity in adulthood and also overweight children are predisposed to later obesity [8]. Obesity is one of the main risk factors for modern chronic diseases [9]. The level of obesity are likely to factor into the consequences of an individual’s overweight/obese condition. The traditional categories (underweight, normal, overweight, and obese) of nutritional status (using body mass index or body fat percentage) need to be re-specified in respect of children’s physical fitness. According to our experiences, besides overweight or obese children, the children who belong to the upper third of the normal nutritional status also often have weaker fitness-related performance than their leaner peers. 

An increasing number of publications have reported a significant relationship between the rate of increase in body dimensions and the higher proportion of body fat content during the circumpubertal years [10,11,12,13,14]. One of the research hypothesis of this paper was that we predicted a taller body height and a more pycnomorphic constitution to correlate with increased relative body fat content. 

Physical fitness is highly dependent on the biological development. The relationships of body composition and maturation have been thoroughly investigated [15,16]. Early maturation is often associated with higher body fat percentage. However, during the developmental process the number of adipocytes increases considerably in 9–11-year-old children [17,18]. The investigation of pre- and peripubertal children in respect of physical fitness and body composition is especially important for effective intervention [19]. 

Though greater body fat percentage generally decreases physical performance [20,21,22], the opposite observation is also available [23]. 

The aim of this study was to analyze the relationship between the various body fat content categories and motor performance in a group of non-athletic pre- and peripubertal schoolboys. Our research questions in this paper were as follows: (1) Are there differences between body height, body weight, body mass index, plastic and metric indexes as indicators of physique by the categorization of body fat percentage? (2) Is there a critical body fat content level in respect of lower performance in physical fitness tests based on the boys’ body fat percentage categories?

## 2. Materials and Methods

A total number of 6919 Hungarian boys (elementary school students in the cities of Győr, Budapest, Szigetszentmiklós, and Nyíregyháza) took part in this investigation. All of the participants were Caucasian. 

According to the recommendations outlined in the Declaration of Helsinki, the subjects were exclusively volunteers. Beyond having obtained the cooperation of the boys and the school staff, written consent was obtained from one of each subject’s parents/guardians before the investigation. All participants and their parents received written information about the goal of the survey and the procedures used. Data management was conducted anonymously. 

The protocol was approved by the university’s institutional review board. The experiments carried out in this study comply with the current laws of Hungary.

Participants were non-athletic children who had a maximum of 1 year of sport-related experience and who trained a maximum of 1–2 times per week. We excluded children with more than the aforementioned amount of physical exercise as well as children who did not report any participation in a sport activity. 

All measurements took part during the curricular physical education classes (2 × 45 min per week). Although the level of habitual physical activity could markedly affect body composition, the extracurricular physical activity of boys was not taken into account as a possible grouping criterion.

The children were grouped by their estimated relative body fat content (Table 1). 

Considering the possible subject number in a given body fat group (especially in the lowest level, G1), the following body fat percentage categories were formed:

G1: estimated body fat percentage is below 8%,

G2: estimated body fat content ranges between 8% and 11.99%,

G3: estimated body fat content ranges between 12% and 15.99%,

G4: estimated body fat content ranges between 16% and 19.99%,

G5: estimated body fat content ranges between 20% and 23.99%,

G6: estimated body fat content ranges between 24% and 27.99%,

G7: estimated body fat content is greater than 28%.

Based on previous experiences [24,25,26], and biological and arithmetic considerations, a finer categorization based on body fat seemed necessary. Ranges of four percentage points were used, because less than that could lead to overly small proportions of subjects in each category, whereas ranges of more than four percentage points could result in a failure to accurately separate subjects. Age groups were created following the suggestions of the International Biological Program [27] following the Helsinki Declaration. Body height and body mass were measured with a stadiometer (model 214, Seca-Bodymorph, Birmingham, UK) to the nearest 0.1 cm and a calibrated scale (model 707, Seca Corporation, Columbia, MD, USA) to the nearest 0.1 kg., respectively. Body height and body mass measures were used to compute the body mass index (BMI) for each child. 

Body composition was assessed by using Lange caliper (Cambridge Scientific Industries Inc., Cambridge, MD, USA) to find the sum of five skinfold sites (triceps, subscapular, abdominal, medial thigh, and calf locations) on the right side of the body. Body fat percentage estimation followed the procedure of Drinkwater and Ross [28]. 

The derived measures (from biacromial distance, lower arm girths, hand circumference, chest width and depth) were taken by Sieber-Hegner (GPM Model 100,113, Geneva, Switzerland) anthropometric set and steel measuring tape (GW-L3595, Budapest, Hungary). Metric (MIX) and plastic (PLX) indexes of Conrad’s growth type [29] were estimated by regression equations [30]. Metric index characterizes the roundness of the trunk in relation to the body height and linearity of the physique (MIX_male_ = 0.1625 × chest depth + 0.13 × chest width − 0.0418 × body height − 0.4245); where a more negative value is considered to be indicative of a leptomorphic condition and a less negative value is considered to be indicative of pycnomorphic condition; plastic index evaluates the level of musculoskeletal development and has strong correlation with the habitual physical activity (PLX = biacromial distance + lower arm girths + hand circumference (cm)).

All measurements were taken three times by skilled anthropometrists, and the average of the measurements was employed in further calculations. The accuracy of the repeated measurements was 1 mm in all measurements, and 0.5 mm in skinfolds. 

According to suggestions provided in previous studies [31,32], four physical fitness tests were used to describe the physical performance capacity, as listed below:

- Standing long jump (SLJ) for the estimation of explosive strength of the lower limbs and leg-trunk-arm coordination. Reading accuracy was 1 cm. The subjects had three trials, and the best score was entered into the statistical analysis.
- Fistball throw (FBT) for the estimation of explosive strength of the shoulder and arm muscles and arm-trunk-leg coordination. Reading accuracy was 0.1 m. The subjects had three trials, and the best score was entered into the statistical analysis (a fistball is a 120g, 8cm diameter ball).
- 30 m dash for the estimation of running speed. Reading accuracy was 0.01 s.
- 1200 m run for the estimation of running endurance (outdoor). Reading accuracy was 1 s.

These four physical fitness tests (which are traditionally used in Hungary) were carried out by physical education teachers. 

### Statistics

Data were analyzed by using Statistica for Windows software (version 11. StatSoft Inc., Tulsa, OK, USA, 2011). As all data were found to be normal, parametric statistical methods were used. All values were expressed as mean ± standard deviation (SD). We analyzed the relationships between body fat percentage and the results of physical fitness tests in the total sample and in the age groups using Pearson correlation. Differences among the means of the created subgroups (based on the relative fat percentage) were tested by one-way ANOVA. In the case of a significant *F*-test, Tukey’s post-hoc tests were used. The level of effective random error was set at 5% in all significance tests (*p* < 0.05).

## 3. Results

Table 1 summarizes the means and standard deviations of percentage body fat by age and fat categories. 

Nine-year-old boys had significantly lower fat percentages than the older boys. Ten-year-old boys had significantly lower fat percentages than 11–12-year-old boys, and 13-year-old boys had significantly lower fat percentages than 11-year-old boys. Ten-year-olds had significantly lower fat percentages than 13-year-old boys in the G2 fat category, and nine-year-old boys had significantly lower fat percentages than 12-year-old boys in the G3 fat category. 

The relationships between body fat percentage and physical fitness tests in the total sample and in the age groups were significant (*p* < 0.05) (Table 2). In respect of FBT, the very low values of correlation coefficients with body fat percentage are statistically significant, but not significant from a biological perspective.

**Figure 1:**
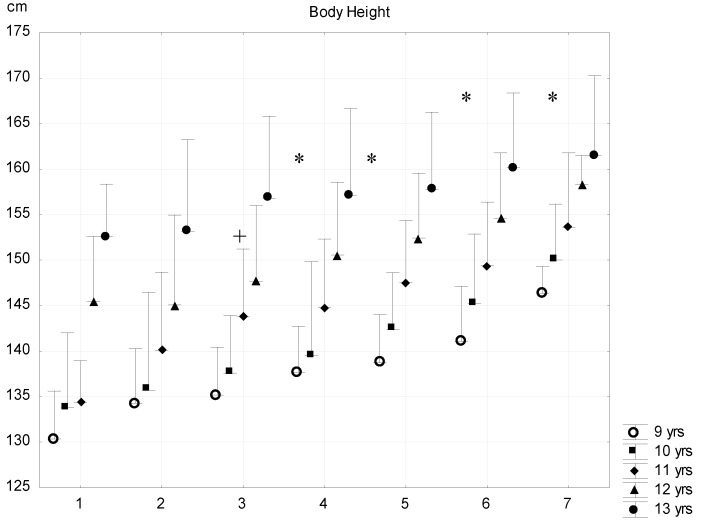
Relationship between body fat percentage and body height (mean + SD). 1—F% < 8.00; 2—F% = 8–11.99; 3—F% = 12.00–15.99; 4—F% = 16.00–19.99; 5—F% = 20.00–23.99; 6—F% = 24.00–27.99; 7—F% > 28; F% body fat percentage; * significant difference among subsequent groups; ^+^ significant difference in certain consecutive groups, *p* < 0.05.

The body height differences were significant among most of seven subgroups compared. The subjects of G1 were shorter than G4–G7, and the subjects of G4 were significantly taller than those of G3, except in regard to the 13-year-old boys. G4–G5 subjects were smaller than G6 subjects, and G6 subjects were significantly smaller than G7 subjects in the 10-year-old age group (Figure 1).

**Figure 2:**
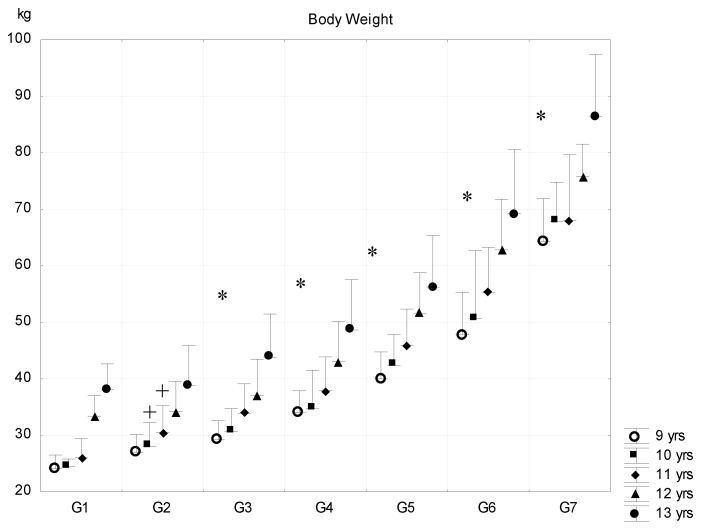
Relationship between body fat percentage and body weight (mean + SD). 1—F% < 8.00; 2—F% = 8–11.99; 3—F% = 12.00–15.99; 4—F% = 16.00–19.99; 5—F% = 20.00–23.99; 6—F% = 24.00–27.99; 7—F% > 28; F% body fat percentage; * significant difference among subsequent groups; ^+^ significant difference in certain consecutive groups, *p* < 0.05.

The differences in body weight among subsequent fat-content groups were significant in most of the cases, except the body weight means of G1–G2 subjects in the 9-year-old and 12–13-year-old age groups (Figure 2).

**Figure 3:**
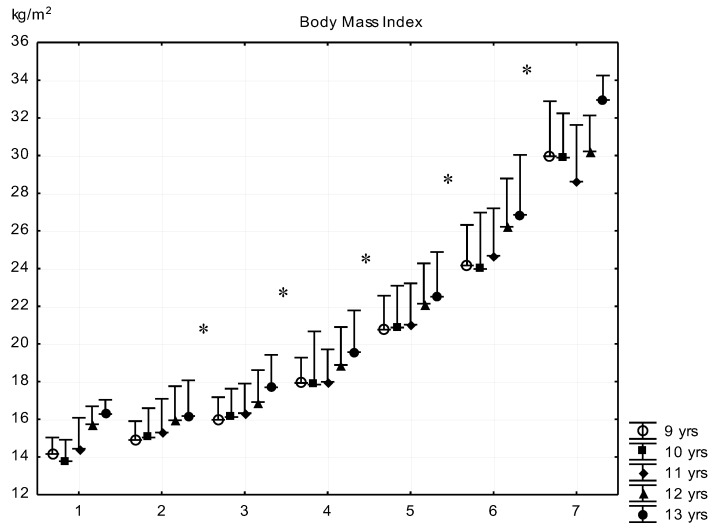
Relationship between body fat percentage and body mass index (mean + SD). 1—F% < 8.00; 2—F% = 8–11.99; 3—F% = 12.00–15.99; 4—F% = 16.00–19.99; 5—F% = 20.00–23.99; 6—F% = 24.00–27.99; 7—F% > 28; F% body fat percentage; * significant difference among subsequent groups, *p* < 0.05.

The means of BMI increased exponentially from G1 to G7, and differences were significant in the subsequent groups except between G1–G2 subgroups (Figure 3). 

**Figure 4:**
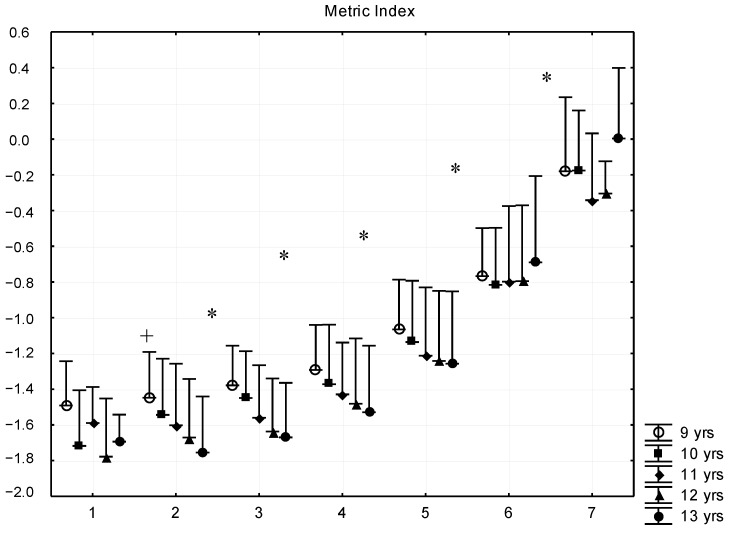
Relationship between body fat percentage and Conrad’s growth type metric index (mean + SD). 1—F% < 8.00; 2—F% = 8–11.99; 3—F% = 12.00–15.99; 4—F% = 16.00–19.99; 5—F% = 20.00–23.99; 6—F% = 24.00–27.99; 7—F% > 28; F% body fat percentage; * significant difference among subsequent groups, ^+^ significant difference in certain consecutive groups, *p* < 0.05.

The metric index (MIX) introduces the roundness of the trunk (component of the physique) between the extremes of leptomorphic and pycnomorphic constitutions. In all age groups, the most leptomorphic boys were the most thin with least body fat, whereas higher body fat percentages were associated with more pycnomorphic constitutions. In all age groups, the metric index was the same for G1, G2, and G3 subgroups, except in the nine-year-old age group, where each subsequent subgroup differed significantly (Figure 4).

**Figure 5:**
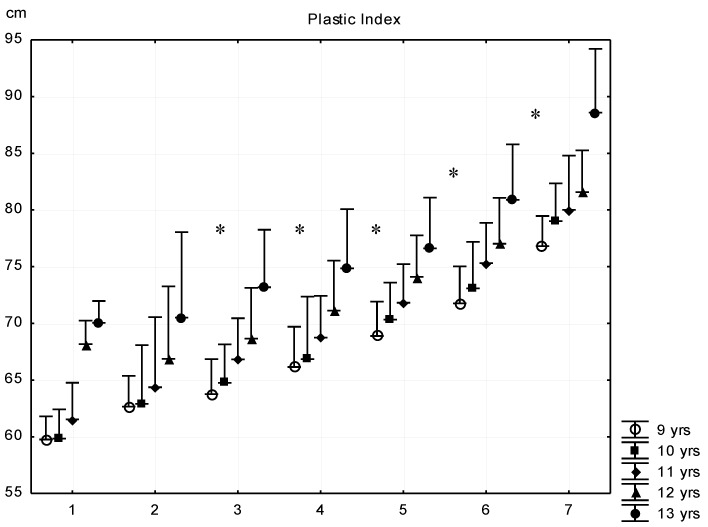
Relationship between body fat percentage and Conrad’s growth type plastic index (mean + SD). 1—F% < 8.00; 2—F% = 8–11.99; 3—F% = 12.00–15.99; 4—F% = 16.00–19.99; 5—F% = 20.00–23.99; 6—F% = 24.00–27.99; 7—F% > 28; F% body fat percentage; * significant difference among subsequent groups, *p* < 0.05.

The plastic index (PLX) describes and evaluates the level of musculoskeletal development. In general, it was observed that subject’s with higher fat content had higher values of robustness. From the three body sizes of plastic index (PLX = biacromial distance + lower arm girths + hand circumference (cm)), the amount of subcutaneous fat plays a role only in the value of the lower arm girths. The forearm has a relatively thin skinfold even in overweight and obese children, thus meaning that it has a slight distorting effect on the value of PLX. All subgroups in all cohorts differed in plastic index, except the G1–G2 subgroups (Figure 5). 

**Figure 6:**
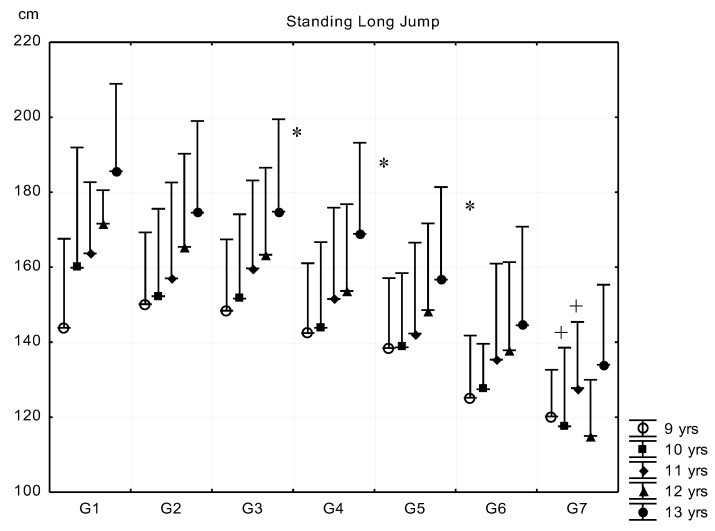
Relationship between body fat percentage and standing long jump (mean + SD). 1—F% < 8.00; 2—F% = 8–11.99; 3—F% = 12.00–15.99; 4—F% = 16.00–19.99; 5—F% = 20.00–23.99; 6—F% = 24.00–27.99; 7—F% > 28; F% body fat percentage; * significant difference among subsequent groups; ^+^ significant difference in certain consecutive groups, *p* < 0.05.

**Figure 7:**
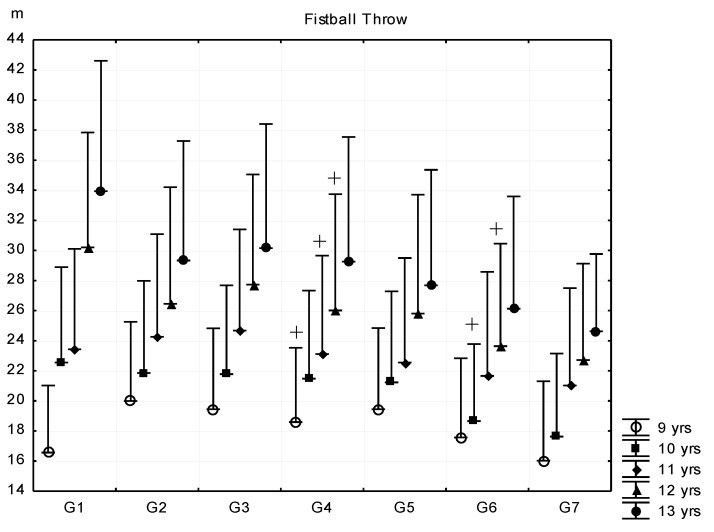
Relationship between body fat percentage and fistball throw (mean + SD). 1—F% < 8.00; 2—F% = 8–11.99; 3—F% = 12.00–15.99; 4—F% = 16.00–19.99; 5—F% = 20.00–23.99; 6—F% = 24.00–27.99; 7—F% > 28; F% body fat percentage; ^+^ significant difference in certain consecutive groups, *p* < 0.05.

**Figure 8:**
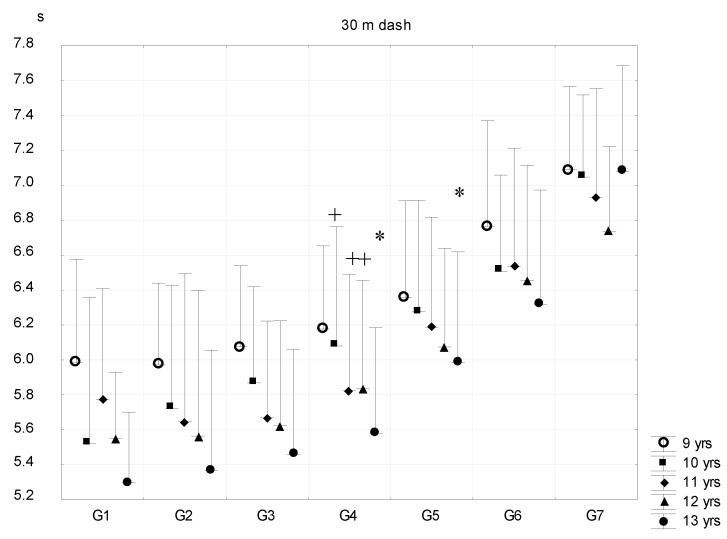
Relationship between body fat percentage and 30 m dash (mean + SD). 1—F% < 8.00; 2—F% = 8–11.99; 3—F% = 12.00–15.99; 4—F% = 16.00–19.99; 5—F% = 20.00–23.99; 6—F% = 24.00–27.99; 7—F% > 28; F% body fat percentage; * significant difference among subsequent groups; ^+^ significant difference in certain consecutive groups, *p* < 0.05.

**Figure 9:**
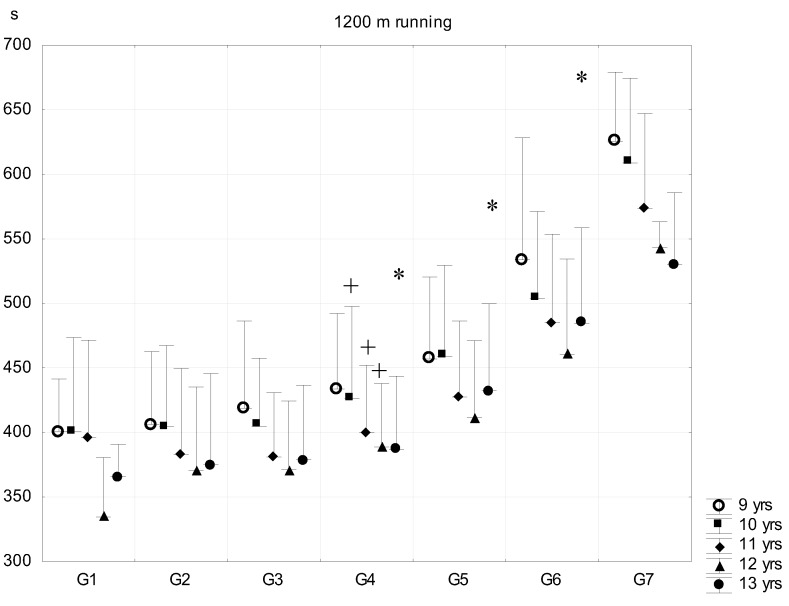
Relationship between body fat percentage and 1200 m running (mean + SD). 1—F% < 8.00; 2—F% = 8–11.99; 3—F% = 12.00–15.99; 4—F% = 16.00–19.99; 5—F% = 20.00–23.99; 6—F% = 24.00–27.99; 7—F% > 28; F% body fat percentage; * significant difference among subsequent groups; ^+^ significant difference in certain consecutive groups, *p* < 0.05.

The means and standard deviations of the physical fitness tests in the five age groups by body fat percentage are demonstrated in Figure 6, Figure 7, Figure 8 and Figure 9. There were significant differences in almost all physical fitness tests of the subgroups. The results of standing long jump test were similar in G1–G3 subgroups in every age group (Figure 6). The means of G1–G3 were significantly higher than those of G4–G7 subgroups, and the differences of standing long jump means were significant in each subsequent group from G4 on, except for G6–G7 subjects in the 9, 12–13-year-old age groups. G7 boys had the shortest distance in SLJ in the 10–11-year-old age group. 

In the following test, more experience and a higher coordination level is needed to perform better. The means of the fistball throw (Figure 7) did not differ among fat percentage categories in the 13-year-old age group. There were only significant differences between G3 and G4–G7 subgroups in the 9, 11-year-old age groups, and G5 and G6 subgroups in the 10- and 12-year-old age groups, who recorded significantly shorter distances. Even the best performance means of the G1–G2 subgroups were moderate and the difference of G1–G2 and G7 was only three to four meters with wide range of standard deviations. Figure 8 and Figure 9 demonstrate the results of the two running tests, namely the 30 m dash and 1200 m running of pre- and peripubertal boys. Significant differences were generally found above the G3 and G4 subgroups. 

The results of the 30 m running test (Figure 8) were similar in G1–G4 subgroups of 9- and 13-year-old age groups—only the children of G5–G7 subgroups (on the borderline of the traditional overweight and obese categories) had significantly poorer performances. From 10 years on, boys with higher than 16–20% body fat content ran significantly slower and subgroups differed significantly. G6 and G7 subgroups did not differ. A higher fat percentage level was associated with lower speed.

Running endurance (1200 m running, Figure 9) in 10- and 11–12-year-old boys differed significantly above G3 in every subsequent subgroups, while in the case of the 9- and 13-year-old boys, body fat percentage was only associated with longer running time when a subject’s body fat percentage was above 20% (i.e., they performed worse). The consistently poor running performance of G4–G7 subgroups was accompanied with a very high variability of performance within those subgroups.

## 4. Discussion

The main goal of this study was to compare some kinanthropometric and physical fitness indicators of prepubertal boys of the same chronological age but of different body fat percentages. We grouped them in narrower fat categories than traditional groupings (the range in subsequent groups was only 4%). However, the low number and high variability of the two extreme groups (G1 and G7) should be taken into account when evaluating the results. A further critical point of the analysis was to select the estimating equation of body fat percentage. We used Drinkwater–Ross’s fat percentage [28] because this widely used model of body composition assesses four fractions (fat, muscle, bone, and residuum); because body weight can be calculated from summing the four fractions, it provides an opportunity to control the accuracy of the estimates. Our results can be compared to the II. Hungarian Growth Study that has used this method as well [13].

There are a number of studies on the kinanthropometric characteristics of overweight and obese children. In our present sample, the fatter children were heavier and taller, which has also been reported in other previous investigations [10,11,33,34]. Body height and body weight differed significantly (a higher body fat percentage was associated with taller stature and larger body weight) in the subsequent fat groups, as well as in children with normal nutritional status under 25% body fat percentage [34].

Associations of fatness and growth type of Hungarian schoolboys have been previously analyzed [35,36,37]. Others [38] investigated children aged 6–11 years and found that the fat pattern was correlated with Conrad’s growth type metric index. They found that in the case of girls, the android vs. gynoid types of body shapes were significantly related with the pycnomorphic vs. leptomorphic types, respectively. In a study of 10-year-old Malaysian boys [37] who were grouped into five fat categories (the differences were 5% of fat), they found that physique changed in parallel with the greater fat content. No other report has found a relationship between metric index and body fat percentage by non-traditional categories (with traditional categories being that a body fat percentage higher than 25% is considered overweight and higher than 30% is considered obese). 

A research team [39] compared groups of nationalities investigating the relationships between growth type and physical performance. They stated that the more pycnomorphic the 13-year-old boys were the poorer motoric capacity they had. 

Growth type differences were significant among most of the seven subgroups of body fat content compared in our study. More body fat content was associated with a more pycnomorphic constitution, and children with a pycnomorphic physique were more susceptible to accumulate excess fat and tended to be overweight and obese. Our present study has confirmed the previous results on a large sample of different age groups.

Conrad’s growth type metric index has been shown to have a remarkable genetic component. Non-athletic Hungarian twins (*n* = 50) were investigated [40], and the heritability of the metric index was 0.78. As the metric index is determined by genetic factors, the pycnomorphic children need special consideration during childhood and puberty to prevent overweight and obesity.

It is well known that the performance of overweight and obese children is generally poorer in motor tests than that of their average fat content peers [26,33,40,41,42]. Indeed, overweight and obese 6- to 10-year-old children showed poorer performance in physical fitness tests in a longitudinal study [43], and in a cross-sectional study as well [44].

Body fat percentage and physical fitness tests correlated significantly, and running tests (1200 m and 30 m) showed higher correlation coefficients with body fat percentage (*r* = 0.37–0.53; *p* < 0.001). Previous studies have shown that having more fat is associated with having lower cardiovascular fitness and explosive strength as well [45,46]. Others [20] have established a strong negative correlation between body fat content and the level of fitness in 7–12-year-old prepubescents. 

In our sample, the running performance (30 m dash, as well as 1200 m running) of the 9- or 13-year-old boys did not differ below 20% body fat content—only the pre- and peripubertal boys with more than 20% body fat percentage had poorer results. In the 9-year-old group, the effect of having had less practice (e.g., the lower level of learning effect) may have modified the results in running performance. Additionally, it may be explained by the various level of maturation: earlier maturation is sometimes associated with higher levels of physical performance, as well as with more fat [15,47]. It seems that in non-athletic peripubertal children that motor performance is limited by more than 20% body fat content. 

## 5. Conclusions

The main finding of the present study was that body fat percentage correlates with the physical fitness of children with normal body fat percentage, even when using merely 4% ranges of fatness for groupings. The lower the relative fat, the better the motor performance was in these pre- and peripubertal boys. Higher body fat percentages were associated with more pycnomorphic body proportions. Based on the results of this study, it seems that the cut-off point for the negative effects of fatness depending on the parameters studied here starts above 16–20% relative body fat content.

We also suggest that 20% relative body fat should be used as the risk level cut-off point for healthy biomedical status in boys [48,49], as opposed to 25% [34]. 

The suggested cut-off point could also be parallel with the significantly poorer physical fitness test performances in the 9–13-year-old boys. In order to build upon the findings between body composition parameters and physical fitness, we suggest that future studies should focus on the genetic background of subjects (i.e., consider the connection between individual physique and the possible predisposition to have higher body fat percentage, especially for cases of extreme pycnomorphic physiques). Doing so could draw our attention to multifactorial variables and further deepen the investigation of intra- and interrelationships between physique, body fat percentage, and performance.

## Figures and Tables

**Table 1:** Body fat percentage categories (mean ± SD) and number of boys in the five age groups.

Sub-Groups	Fat%	*n* (9 Years)	*n* (10 Years)	*n* (11 Years)	*n* (12 Years)	*n* (13 Years)
		(Fat% Mean ± SD)				
G1	<8	7 (7.1 ± 0.8)	8 (6.9 ± 0.9)	9 (6.5 ± 0.4)	5 (7.2 ± 0.3)	5 (7.2 ± 0.4)
G2	8–11.99	181 (10.5 ± 1.1)	223 (10.3 ± 1.4)	139 (10.4 ± 1.5)	132 (10.5 ± 1.5)	121 (10.7 ± 1.5)
G3	12–15.99	399 (13.9 ± 1.1)	515 (14.0 ± 1.1)	412 (14.1 ± 1.1)	443 (14.2 ± 1.0)	419 (14.1 ± 1.1)
G4	16–19.99	264 (17.9 ± 1.2)	391 (17.8 ± 3.9)	400 (17.9 ± 1.1)	387 (17.9 ± 1.1)	344 (17.7 ± 1.1)
G5	20–23.99	184 (21.9 ± 1.1)	322 (22.0 ± 1.2)	302 (22.0 ± 1.2)	270 (21.8 ± 1.2)	218 (21.9 ± 1.2)
G6	24–27.99	72 (25.5 ± 1.1)	162 (25.4 ± 1.1)	210 (25.7 ± 1.1)	187 (25.5 ± 0.9)	122 (25.5 ± 1.1)
G7	>28	9 (28.8 ± 0.7)	22 (29.3 ± 1.0)	24 (29.0 ± 1.2)	5 (28.2 ± 0.2)	5 (29.0 ± 0.7)
		1116 (16.5 ± 4.6)	1643 (17.3 ± 4.9)	1496 (18.2 ± 5.0)	1429 (17.8 ± 4.6)	1235 (17.3 ± 4.5)
Total		*N* = 6919 (17.5 ± 4.8)				

**Table 2:** Correlation pattern between body fat percentage and physical fitness tests.

*r*	Fistball Throw (m)	Standing Long Jump (cm)	30 m Dash (m/s)	1200 m Running (m/s)
Fat%—total sample	−0.13 ***	−0.34 ***	−0.39 ***	−0.44 ***
Fat%—9 years	−0.10 *	−0.33 ***	−0.37 ***	−0.42 ***
Fat%—10 years	−0.14 ***	−0.38 ***	−0.42 ***	−0.48 ***
Fat%—11 years	−0.17 ***	−0.35 ***	−0.42 ***	−0.52 ***
Fat%—12 years	−0.15 ***	−0.36 ***	−0.43 ***	−0.45 ***
Fat%—13 years	−0.16 ***	−0.39 ***	−0.44 ***	−0.47 ***

Most anthropometric variables and physical fitness test results showed a similar tendency in all subgroups by age in respect of fat percentage categories. * *p* < 0.05; *** *p* < 0.000.
